# Living with disabled children in Malawi: Challenges and rewards

**DOI:** 10.4102/ajod.v5i1.254

**Published:** 2016-08-24

**Authors:** Grete Barlindhaug, Eric Umar, Margaret Wazakili, Nina Emaus

**Affiliations:** 1 Department of Health and Care Sciences, Faculty of Health Sciences, UiT the Arctic University of Norway, Norway; 2 Department of Community Health, University of Malawi, Blantyre, Malawi

## Abstract

**Background:**

Rehabilitation personnel need to be sensitive to the cultural aspects that constitute the environment of a disabled child’s family life.

**Objectives:**

The aim of this study was to gain insight on how families experience parenting of disabled children and how the families experience the support provided by the rehabilitation system in Malawi.

**Method:**

An anthropological field study combining interviews and observations was conducted in a rural district of Malawi in 2011. Permission was granted to follow four families, and this study presents the stories of two families, whose children have severe disabilities. We used phenomenological and narrative analyses to make sense of the stories.

**Results:**

The findings indicate that families with disabled children invest time and emphasise care for their disabled children. They feel enriched by their experience despite challenging situations with little support from the rehabilitation services. High standards of care demonstrating positive and moral attitudes have earned these families respect in their communities. Storytelling has created an opportunity for the families to understand and interpret their challenging situation with inherent contextual meaning.

**Conclusion:**

This study shows that families with disabled children draw on cultural and structural strengths that rehabilitation professionals should be aware of in their support to mothers and other caregivers of children with disabilities.

## Introduction

The World Health Organization (WHO) report on disability underlines how the person’s environment influences the experience and extent of disability (WHO 2011). The cultural aspect constitutes part of the environment of the family life with a disabled child. Within medical anthropology and social and health sciences, this aspect is described from different perspectives. Over the years, research has emphasised the burden and the emotional distress associated with parenting of children with disabilities (Dambi, Jelsma & Mlambo 2015). Parents experience the negative consequences of stigma and socio-structural constraints (Green 2003, 2007; McKeever & Miller 2004; Ryan & Runswick-Cole 2008). However, recent research also indicates that a narrow focus on emotional anguish is far too simplistic. Such research has shown that many parents of children with disabilities identify several positive aspects of their experience (Green 2002, 2003, 2007; Heiman 2002; McKeever & Miller 2004; Milo 1997; Ryan & Runswick-Cole 2008; Traustadottir 1991). These studies suggest that caring for a disabled child reinforces strong feelings of love (Traustadottir 1991), pride, respect and value, growing courage and emotional strength (Green 2002, 2003), personal growth (Milo 1997), strong social and family bond and deeper relationships with friends and family (Ryan & Runswick-Cole 2008). Parents feel acknowledged as competent people with lives enriched by their children (Green 2007; Heiman 2002). Therefore, the existing literature concerning parenting of disabled children shows conflicting results.

To create a positive, good and meaningful (re)habilitation process, it is important that rehabilitation personnel and social workers appreciate and acknowledge the efforts of the families that strive to create a functional daily living environment for their disabled children. Encouragement and support to the family that promotes existential hope in the present situation and for the future is of immense importance (Heiman 2002; Mattingly 2010; McKeever & Miller 2004; Milo 1997; O’Toole & Maison-Halls 1994), especially where resources are limited. Malawi is a country of 15 million inhabitants and one of the poorest countries in the world. Health resources are scarce, and there is an acute shortage of rehabilitation personnel. Families with disabled children cope with life without the network and support that is usually provided by the state in many western countries. This study illuminates multiple aspects, including the physical and emotional wellbeing of two families with their severely disabled children in Malawi. Thus, the aim of this study was to gain insight on how families experience parenting of disabled children and to gain insight into how the families experience the support provided by the rehabilitation system in Malawi.

## Theoretical perspective

In the present study, we have used an analysis based on a narrative phenomenology of practice inspired by Cheryl Mattingly (2010), to understand how families with disabled children manage their lives and overcome daily challenges in meaningful ways despite their experience of despair. In the analysis of practice and reality, Mattingly (2010) brings together multiple perspectives including a phenomenological approach as well as an analysis of how the social structures influence our lives. She explores the relationships between subjectivity and the social and political orders and describes how this relationship is influencing and shaping our practice. Mattingly (2010) offers a theory of practice that illuminates life at multiple levels, seeing it through the prisms of the personal as well as the structural forms. In her analysis, she highlights the small dramas of ordinary life, the particularity of events and personal agency. With a focus on the agents themselves, she examines their efforts in situational settings, treating the larger macrostructures as powerful cultural resources that influence persons and situations without determining their actions, experience and deliberations.

The narrative phenomenology described by Mattingly (2010) uses concepts such as motive, character, plot and scene to analyse person-centred practice. These ways of analysing agency and intention in practical life were initially offered by Kenneth Burke as far back as 1964 (Mattingly 2010). Into this, Victor Turner (1974, 1987) included the notion of social drama. In the latest decades, narrative has emerged as a key term in the analyses of the temporality of lived experience, of actions, suffering and thoughts. Narratives contribute to an understanding of social life and experience of time as the unfolding of events. Mattingly uses the terms drama and narrative almost interchangeably; drama is used to underscore the eventfulness of social action and narrative to describe the temporal complexity of situations and practice.

Michael Jackson is another anthropologist who emphasises the inter-subjectivity of dramas in the relationships between the one and the many, the particular and the universal (Jackson 1998). He describes inter-subjectivity as a forceful field charged with energy and driven by need. People need to belong to and engage effectively in a world of others with a need to have some saying and a sense of making a difference. All the time, individuals have to struggle for a balance between one’s own needs and needs of others, between the world one calls one’s own and a world of not self or other. The relationships between persons are of utmost importance (Jackson 1998, 2002). Jackson underscores the importance of seeing life dramas through the relationship between microcosm and macrocosm, between the visible and invisible, the familiar and the foreign, and the living and the dead (Jackson 2002). Jackson also describes the inter-subjective life dramas as a sense of agency, where the most compelling is the human need to imagine that one’s life belongs to a matrix greater than oneself, and in this sphere of greater being one’s own actions and words matter and make a difference (Jackson 1998). What matters in life events is how the inter-subjective dramas, the stories enable us to regain some purpose and control over the events that confound us, over disempowering circumstances, giving us the faith that the world is within our grasp (Jackson 1998).

The present study takes a person-centred approach with an interest in the aspects of hope in people’s life struggles. Inspired by Mattingly, we want to examine hope in a practice that is contested, dynamic and hybrid (Mattingly 2010). Based on Jackson’s theory, we want to examine the life on the ground in all its local and personal immediacy and drama (Jackson 1998). For the analyses of how the social structure informs people’s actions, we also lean on Pierre Bourdieu’s theory of practice, which helps to understand the dialectic between objective structures and concrete action and interaction. His analytical concept of habitus focuses on the dynamic relations between the social and the individual. Habitus encompasses the incorporation of social structures in individuals and groups as dispositions and tendencies to perceive, move and act in socially acceptable and adjusted ways. Habitus is acquired through participation in everyday activities and relations and is influenced by the cultural aspects surrounding our lives (Bourdieu 1990; Bourdieu & Wacquant 1995).

## Methods

### Research setting and study design

The material developed during a 4.5-month-long anthropological field study conducted by the first author (G.B.) in a rural district in Malawi. The University of Malawi, College of Medicine Research Ethics Committee in Blantyre granted ethical approval for the study. Families with disabled children were contacted through the outreach rehabilitation programme in Blantyre where they were registered. Oral and written information about the study was given in *Chichewa* (local language) by the Malawian co-researcher (E.U.) to the families that agreed to participate in the study, and they were informed that their participation was voluntary. The families accepted orally, and the researchers were welcomed to follow four families in their daily life. In this study, we present the stories of two of the families, whose children have severe disabilities. The other two families’ children had minor disabilities with less challenging everyday life. Each family in this study was followed 5 days a week over a period of 1 month through everyday family life and village activities. This allowed a direct access to the two families’ practices, interpretations and experiences in their natural setting. Wollcott (2008) describes fieldwork as a special way of viewing human social behaviour. He emphasises that being in the field over time gives a researcher the opportunity to become gradually closer to people’s concepts of life and to understand how their worldview influences life’s projects and provides meaning to events and life experiences. Because the researcher (G.B.) did not know *Chichewa*, a female local research assistant without any formal qualifications followed the field study and assisted with daily conversations with the families and asked questions about actual life events. At the end of each day, the researcher and the assistant summed up the happenings, wrote down the field notes and prepared for the next day. In addition to this, semi-structured interviews were conducted by the Malawian co-researcher (E.U.) to complement the observational data. In this study, we only utilise data from one semi-structured interview in addition to the field notes.

### Study participants

The two families with severely disabled children who require a great deal of help and care both live in the same village and depend on small-scale farming. The names of the children and their family are constructed to secure anonymity. ‘Anna’ is 11 years old. She lives with her parents, one older sister and brother and three younger siblings. Two of her brothers and one sister have moved out. Anna contracted malaria when she was 7 months old and suffered brain damage with consequent motor and cognitive delay. She moves around by crawling and can stand for short periods while holding on to objects. She has minimal expressive language but understands what people say to her. An interview was conducted with Anna’s mother after the end of the fieldwork, and these data are integrated in the empirical description. Josephine is 9 years old and lives with her grandmother, five aunts, two cousins and two other members of the extended family. Josephine was born with brain damage, which is classified as cerebral palsy. She is in total need of help for all her daily activities.

### Analyses

Written notes from the observations and conversations were taken throughout the fieldwork. The formal interview with Anna’s mother was transcribed in *Chichewa* and translated to English by the Malawian co-researcher (E.U.). Inspired by the outlined theoretical perspective, analysis of the observational and transcribed data was based on content analysis (Polit & Beck 2012) inspired by a narrative approach. The analysis of the text data included coding, categorisation of the coded topics, identification of basic units and main themes and meaning construction in a circular process. Through this process and the narrative approach, the results emerged as representative stories for the everyday life of the two families under study. Typical everyday activities and social life emerged as central themes in the analyses, so did the story about how the child contracted the disease and the encounter with the healthcare system, and then finally, how the families are coping and their thoughts about life with a disabled child. Based on the analyses, the results are presented as stories for each family separately.

## Results

### Empirical description: Anna

#### Everyday activities

When we arrive at the family’s house in the morning, we are greeted by Anna’s mother who places a mat under the tree and we sit down. Anna comes crawling from behind the house and sits next to us. The oldest sister and her baby also sit down on the mat and greet us. Soon there is an eager chat. It does not take long before we are surrounded by other children. The sister leaves the baby with us and goes to do some dish washing. She needs more water, and together with Anna, we follow her to the water pump where Anna pumps the water because she really loves the activity. The sister carries the water bucket on her head to the house. After some time, Anna’s father comes from the mountain bringing firewood on his bike. He sits down beside the house and works on a new mat on which they dry the corn. The sister makes some porridge for the baby and other small children in the household. Anna likes porridge as well and is given what is left over by the children. Then Anna sits naked on a stone in the backyard, while the mother gives her a bath and leaves her to dry in the sun before changing into clean clothes. After that, the mother supports Anna under her arms and lets her ‘walk’ a few meters to the wall of the house, and sits her down. We are all served pumpkin.

The mother breaks some little branches from a tree in the backyard and sits on the mat while talking to us, and we help her pick small leaves off the branches. She uses these leaves and some tomatoes to make a vegetable dish for lunch. She has also bought some peanuts. We help in shelling before she uses a mortar and pestle to crush them into a powder that she adds to a vegetable dish to improve flavour. Anna helps with the crushing of the peanuts. Then, it is time to move to the kitchen area in the backyard to make *nsima* (thick porridge). This is the main meal served with the vegetable dish. Before serving the lunch, Anna’s mother goes to the mosque for afternoon prayers.

Sitting on the mat, preparing vegetables, shelling corn from the cob or just talking, lots of people are passing and sitting down for a chat, after a polite and appropriate greeting. Anna’s grandmother often drops in on short visits while her aunt makes longer visits, bringing her own vegetables or corn to shell off the cob. Some women come to greet us, and they sit down eager to share their stories and hear news from friends. Other people also pass a short greeting and sometimes stop to ask questions or take part in the storytelling. Women carrying buckets on their heads pass by selling tomatoes, beans, vegetables and small fish. Men and young boys come to see the father or the son, and they also greet us, often by hand-shake. The children sit listening to the adults or play with home-made balls, singing, running around or roasting some corn to eat. Anna knows some of the songs and tries to sing as well, and when the other children run away, she eagerly follows crawling on all fours.

During sowing or harvesting season, Anna’s mother works in the field because that is her livelihood. When Anna was young, her mother used to carry her on the back, but now she is older and heavier, so she stays at home while everyone goes to school or to the garden. The brother, niece and mother often take turns to look after Anna at the house. Anna’s mother told us that she is afraid that her daughter might try to follow her to the fields where they cross a dangerous creek, which Anna may not manage on her fours. Sometimes Anna has followed after her mother, and other people have alerted her to bring her daughter back home. The mother is also concerned about what may happen when Anna reaches puberty, because she will not be able to clean herself after her menses. In addition, if Anna stays alone at home when the other children are at school and the adults in the fields, she may be at great risk of being sexually abused. If that should happen, Anna may not be able to tell anybody about the incident or the culprit. Such concerns would be averted if Anna had a tricycle, as she could then accompany other children to school or the adults to the field.

#### Encounter with the healthcare/rehabilitation system

Anna’s mother tells us her daughter was born healthy and normal. When she was 7 months old, she was sleeping inside the house while the mother did the chores outside. She heard a cry and went in to find the baby unconscious with foam around her mouth. At the time, the family lived in a district away from close family and friends. The mother called her neighbour who suggested that they should go to the hospital. At the hospital, the baby was in coma for 1 week, and she stayed a full month in hospital. The doctor said that Anna had cerebral malaria and epilepsy, but comforted the mother that her daughter would be all right and compared her with other children who had died. After several medications, Anna recovered, and they went home. As time went on, it became obvious that Anna had been severely affected. She could not sit and the condition had affected her speech and ability to eat, she could not see properly and her head was growing bigger.

After discharge, the family returned to the home village and visited the local hospital where they met a rehabilitation technician on a regular basis. Anna was provided with splints to straighten her legs for standing during the day and to keep the knees straight at night. The mother was encouraged to stand her daughter against the wall. After some time, she did not get any more plasters, and the standing exercise stopped. Later, the rehabilitation technician asked the mother to dig a small pit to act as splints, in which Anna would stand well supported by the walls of the pit. They did not do any exercises at the hospital. Instead, they showed her how to do the exercises and she has done everything as she was told, to the best of her ability, and in a way, therapy has been successful. Eventually, Anna learnt to sit and crawl on her fours. However, the mother is disappointed that her daughter did not get the tricycle that she was promised: ‘They promised me this bike for the girl, and every time I went to the hospital the bike was not ready’. In due course, the mother received some money from a local member of parliament and went to the tricycle workshop. There were none available for sale and neither were there hand and knee pads to protect her during crawling. Anna has continued to have epileptic seizures, for which the mother took her to a traditional healer on several occasions without success. However, hospital medication had reduced the number and intensity of seizures to the extent that these only appeared by each new moon. At the time of this interview, the family had run out of drugs and the number of Anna’s seizures had increased again.

#### Life with a disabled daughter

Anna’s mother explains that her life has been greatly affected since her daughter’s illness. She continually wonders about what had happened that could explain this terrible occurrence, especially considering that all her children were born well and healthy. Why should this happen to just this child? She has not found any answer, and she has accepted that this must be the will of God. ‘It has been a burden to me and affects me up until today because my child is unable to walk’. Although Anna’s mother had hopes that her daughter would be able to walk, she nevertheless loves this daughter so much, because God might curse her if she does not. ‘I need to take care of this child and love her as much as I can until I see what God has provided’. The whole family supports Anna, and the mother of five has told all her children to love their sister just the way they love themselves, because this is what God has given to them. If they turn their back to their sister, God will not be happy with them. She encourages them not to ignore but to love Anna even after she herself is dead. The siblings know how to take care of her, so the mother is convinced that when she dies, Anna will still be taken good care of.

## Empirical description: Josephine

### Everyday activities

When we arrive early in the morning, Josephine’s grandmother and her entire household are working in the fields before the sun is too hot, while another guardian stays at home looking after Josephine who is still asleep in the yard at the back of the house. The guardian greets us and takes Josephine onto her lap. When Josephine’s grandma returns, she greets us and starts to make breakfast for her granddaughter and two other grandchildren; tea with sugar and milk powder. The grandmother takes Josephine onto her lap and pours some tea into her mouth while in half-lying position. Most of it comes out because Josephine has problems with swallowing. After breakfast, grandmother helps Josephine to brush her teeth and take off the wet clothes. A piece of cloth is wrapped around Josephine, and we follow them to the front of the house where we all sit down on a mat under a big tree. In a right-size plastic bath tub, Josephine sits with her legs crossed leaning against the tub wall. The grandmother washes her thoroughly, dries her and applies Vaseline on her skin lay before changing her into clean, nice clothes.

After her bath, Josephine is laid on the mat and surrounded by children in the family and neighbourhood who play around her. The grandmother sits with us for a short while and then leaves to do some household chores such as laundry and house cleaning. During this time, we look after Josephine and a family member joins us on the mat while preparing vegetables or just talking. In this household, most of the time is spent cooking, doing dishes or looking after small children. When Josephine is tired or bored, she makes protesting noises, and her grandmother attends to her immediately. We observe that Josephine is throwing up again when she is helped to sit up. The dress gets wet, and her grandmother lays her down on the mat and leaves to find a clean dress to replace the wet one. After a short time, the grandmother continues with her housework while Josephine is placed in a wooden chair. She sits in her chair for a short while trying to hold her head up to look at some children playing. It is hard for her; she gets tired and starts whining. Then, she is placed on the mat, but Josephine is not happy until her grandmother takes her on the back and then she smiles with satisfaction.

Josephine is 8 years old and it has become increasingly impossible for the grandmother to carry the big girl on her back for a long time. For this reason, Josephine is placed on the mat where some children and adults always gather and she accepts to be in the company of the others for a long time. But after some time, she pees and wets her clothes again. The grandmother comes back and changes her into dry, clean clothes. Josephine’s grandmother does a lot of laundry on a daily basis and she complains about the quantity and the costs of the soap. While sitting on the mat, we observe that people are passing all the time on their way to the water pump and to visit friends or families. Most of them stop and greet us as visitors and the women who are there with us, before they continue on their mission. Some women sit down for a short time and some talk about food is exchanged. Others only greet and sit down silently.

### The encounter with the healthcare/rehabilitation system

Josephine’s mother died in pregnancy at the district hospital just before giving birth. The baby was delivered by a caesarean section, and the grandmother brought the baby back home to the family where she already had five girls and the youngest was 6 months old. It turned out that baby Josephine had suffered massive brain damage and developed cerebral palsy. At the hospital, the family was referred to the rehabilitation unit. Once a month, they attended treatment and learning sessions about how to handle and exercise the baby. The grandmother also took Josephine to the city to a private rehabilitation centre for physiotherapy for 3 months every year over 3 years. A special wooden chair and a standing frame were made at the workshop, and the grandparents paid for it. The private centre closed down, and a monthly visit to the local rehabilitation unit is the only treatment option available to the family. The grandmother also visited a Chinese doctor who gave her some vitamins and minerals and said it would give Josephine more energy and improve her cognitive skills. She was also taken to the district hospital because of spasms and stomach pain. The doctor showed the grandmother how to gently massage the hard stomach and press the air out. She has done so, and it helps. In addition, she told us that Josephine is easier to handle and less stiff than before. On the whole, we were told a lot about the help Josephine has received from the healthcare system. This grandmother does not focus on what they have not been provided, but underlines the fact that most activities are done on her own initiative and at a very high financial cost to the family.

### Life with a disabled granddaughter

Josephine is in need of total help in all daily activities and her grandmother provides such help and care. Seldom does the grandmother receive help from her sister or the eldest daughter. In addition to her daily activities, the grandmother also carries out exercises with Josephine. The exercises are mostly passive movements of the joints, some active side rolling, active leg movements and head lifting from the mat and short periods of sitting in the chair or standing in the standing frame. When Josephine is situated on the mat, in the chair or in the standing frame, with other children around, the grandmother can do some house work. She underlines that a lot of laundry is needed and that she uses a lot of money to buy laundry soap. Despite this, the grandmother shows a deep attachment and great affection for Josephine. She is aware of the brain damage that occurred at delivery, and she believes that God has given them this child. She is always there for the child when other family members have to leave the house for shopping or other activities.

## Ethical considerations

The study was conducted in accordance with relevant national and international guidelines. The University of Malawi, College of Medicine Research Ethics Committee in Blantyre granted ethical approval for the study 19.07.2010 (P.09/08/6/99). Families were contacted through the outreach rehabilitation programme where they were registered. Oral and written information about the study was given in Chichewa (local language) to the families that agreed to participate in the study, and they were informed that their participation was voluntary.

## Discussion

### The struggle to create a life worth living

When a family gets a disabled child, they experience initial shock and despair (Green 2007; Mattingly 2010). The child is different from what they expected. In a way, they are plunged into a new and unknown situation and have to struggle to navigate through this new life, at a personal, family and community level (Green 2002, 2007; Read 1991). This experience is described in a similar way, regardless of where in the world the family comes from. The narratives presented here from Malawi illustrate how the families after the initial shock cope with challenges, worries as well as love and try their utmost to create meaning and hope under difficult circumstances (Ficher & Goodley 2007; Heiman 2002; Landsman 2003; McKeever & Miller 2004; McLaughlin 2006).

Unity and solidarity help Anna’s family to navigate through life not only practically but also emotionally, as seen through their smiles and good moods, the smooth division of work and the openness to friends and neighbours. In this poor village community, the family is the organising structure of the society and a valuable resource in managing the new life with a disabled child. The small family is working hard together and they are supported by the larger and extended family and well respected in the community.

Josephine’s family seems to be in a different situation, where all female members of the family cooperate when it comes to execution of daily tasks, except that the grandmother takes full responsibility for her disabled granddaughter. The grandfather is absent from the family because he lives in South Africa, but he has sent money home over several years. Their family house is bigger than is usual in the village, it has a sitting room with furniture, TV and radio, none of which we saw in the neighbouring houses. They also have a solar panel that generates electricity to charge mobile phones at a small fee for people in the village. Despite these extra resources, the grandmother has obviously an extra burden of caring for Josephine.

Families with a disabled child are often different from other families and as such carry a social stigma that distinguishes them from other members of the society (Goffman 1968). For the families we met during this field work, it seems important to be different in a good way. Thereby, they gain a positive distinction, both by their participation in society and in the caring of the disabled child. The families want to avoid stigmatisation and to live good, moral lives seems extremely motivating. The stigmatisation seems to be a strain to the mothers and the families, but coping well seems to lessen the stigma (Green 2003). The stories of everyday life illustrate what is at stake for people in the village, and the families work hard to live up to what is highly valued: the respect of others. They work hard to care for the family, keep the house and yard nice and clean, prepare enough and tasty food and dress all family members in clean clothes after a daily bath. The mother/grandmother cares particularly about the welfare of the disabled child. This may be interpreted as a personal strategy to be positively regarded in the society and to diminish the stigmatisation. This seems most striking in Josephine’s situation. The internalised, cultural knowledge and the habits seem to guide the mother and the whole family in their conduct of everyday life. Their own and other’s actions may be understood as meaningful contributions in their inter-subjective drama (Jackson 1998).

The stories also tell us that in addition to getting most resources out of their own property, someone in the family has to be employed to get money to be able to buy what is needed. The fathers have to find gainful employment outside the village, either in the nearest township or in another country, here exemplified by South Africa. The two fathers in the families in question have chosen different strategies. The father in Anna’s family stays in the village supporting the family in daily practical and social life, working as a night guard in the township to provide extra money for the family. The father in Josephine’s family lives in South Africa and supports the family from a long distance. This extra support is vital for people to live culturally and highly valued lives that are important to families of children with disabilities.

Everyday life stories also tell us that Anna’s family gives great priority to fulfil the social expectations of community life. We can see this as a strategic personal agency to act as a normal family. Participation in funerals and the celebration 3 days after a funeral is highly appreciated, and Anna’s mother and other family members take part in these social occurrences. Sharing time with friends, listening to their stories, showing interest, giving personal opinion and sharing knowledge is also valued. In Josephine’s family, the grandmother spends less time socialising with other villagers, but gives priority to providing nice clean clothes and good daily care for her disabled granddaughter, all of which are very time consuming. In addition, she values her achievements and material possessions such as a big house, furniture and the solar panel. In this way, the family strives to find a balance in meeting their cultural and personal needs and those of others (Jackson 1998).The stories also tell us about the lively social activity outside Anna’s family house. The greetings are respectful and Anna’s family indicates an inclusiveness more than what is usually observed in thisv illage. This openness and continuous presence can be interpreted as a strategy of personal agency to be an efficient member of society (Mattingly 2010). It displays an inter-subjective drama of willingness to engage effectively in a world of others to have something to say and get the sense of making a difference (Jackson 1998).

### Hopes and dreams for the future

The encounters with the healthcare and rehabilitation system illustrate how little help these families receive from organised services and how they are left alone to cope with their disabled children. Anna’s mother explains how her daughter’s illness and the subsequent missing motor and sensory development have been such a constant worry to her all the time. The storytelling about everything that has happened shows how the mother sees life with a disabled daughter as a journey from the past into the future – what happened and what might be. Through this process, she creates hope that Anna will be healthy and develop in a normal way, learn to walk, and have a tricycle so that she can move around the village. She trusts that the older children will take care of Anna when the mother is no longer around.

The plot of the story tells how Anna’s mother has done everything that was expected of her to the utmost of her ability and that it was fruitful in as far as her daughter reached some functional milestones. The only setback was that the delivery of bandages for applying splints to Anna’s weak legs came to an end and that she did not got the tricycle she was promised. This shows how the story can be a tool for making moral judgement through the configuring of the plot (Mattingly 2010). The mother underlines all her efforts and achievements in the rehabilitation process. This seems to keep her self-esteem up (Ficher & Goodley 2007) and the community will judge her efforts as a highly moral part of motherhood (Traustadottir 1991). At the same time, she underlines who did not keep their promises and who is to blame for her daughter’s lack of walking function. Josephine’s grandmother does not express any complaints about the healthcare and rehabilitation services, but her story reinforces her own initiative and action when it comes to health and rehabilitation and how much money she has spent, which shows the agency of high morality.

Storytelling may also be interpreted as a process of understanding and coming to terms with what has happened and why. It helps in the creation of meaning and hope in a challenging situation without any inherent meaning. Anna’s mother tells about her long time of pondering about the reasons why her daughter fell ill. She could not find any special incident that could explain why this tragedy happened to this special child, but she has accepted it now as the will of God. She tells how she and the family have to care for Anna and love her the way God expects and ensure to obtain a reward in turn. Josephine’s grandmother has come to the same conclusion that her granddaughter is a gift from God that has to be cared for in the best way possible and she takes this mission seriously. These stories tell us how the mothers let their life dramas exceed the relations between private and public realms. They embrace the relationship between the visible and the invisible, the familiar and the foreign by taking the life dramas to the realms between microcosm and macrocosm (Jackson 2002). The existential experiences of their life belonging to a matrix greater than themselves can be seen as a representation of inter-subjective life dramas where the most compelling is that in this sphere of greater being one’s own actions and words matter and make a difference (Jackson 2002). The inter-subjective dramas and the plots give the mothers possibilities to regain influence and control of the life situation, providing faith that the world is within their grasp.

### Summary of findings

The process of following families with disabled children in Malawi underlines how such families gain respect and added value in society despite the challenges associated with everyday life. The mother’s and the grandmother’s daily practices seem strongly motivated by achievement of favourable inter-subjective relations in their particular cultural, social and economic environment. The practical life stories tell how by self-sacrifice they take care of disabled children and gain respect from others as well as create meaning and hope in their own lives. Similar findings have also been reported from other studies around the world (Case 2000; Dambi *et al*. 2015; Ficher & Goodley 2007; Green 2003; Landsman 2003; Mattingly 2010; McKeever & Miller 2004; McLaughlin 2006; Milo 1997; Ryan & Runswick-Cole 2008; Traustadottir 1991). In line with our findings, other studies have shown that families highly value rehabilitation personnel who understand their thoughts and meaning-making processes (Goodley & Tregaskis 2006; Heiman 2002; Mattingly 2010; McKeever & Miller 2004; Milo 1997; O’Toole & Maison-Halls 1994). It is our hope that this study will contribute to the understanding of how the personal everyday practices of families and especially mothers of disabled children are informed by cultural and structural forms. The strength of this study lies in its design; an anthropological fieldwork study comprising observations during everyday life activities over several months combined with in-depth interviews. The limitation is the number of families followed as an extended fieldwork could have provided insights into other challenges encountered by other families with disabled children. Language was also an obstacle during the fieldwork providing a challenge for conversations except everyday small talk. However, the local research assistant was a great help throughout the fieldwork and we are greatly indebted to her.

## Conclusion

Despite heavy challenges, families with disabled children draw on cultural and structural strengths to master their everyday activities, maintain hope and construct meaning. Rehabilitation professionals and social workers should draw on these insights to enhance the care and support that the mothers and other caregivers provide to children with disabilities. In collaboration with the families, the professionals in question should set realistic and achievable goals.
